# Global Patterns in Post-Dispersal Seed Removal by Invertebrates and Vertebrates

**DOI:** 10.1371/journal.pone.0091256

**Published:** 2014-03-11

**Authors:** Begoña Peco, Shawn W. Laffan, Angela T. Moles

**Affiliations:** 1 Terrestrial Ecology Group, Departamento Interuniversitario de Ecología, Facultad de Ciencias, Universidad Autónoma de Madrid, Darwin s/n, Cantoblanco, Madrid, Spain; 2 School of Biological, Earth and Environmental Sciences, the University of New South Wales, Sydney, Australia; 3 Evolution and Ecology Research Centre, School of Biological, Earth and Environmental Sciences, the University of New South Wales, Sydney, Australia; Dauphin Island Sea Lab, United States of America

## Abstract

It is commonly accepted that species interactions such as granivory are more intense in the tropics. However, this has rarely been tested. A global dataset of post-dispersal seed removal by invertebrates and vertebrates for 79 native plant species from semi-natural and natural terrestrial habitats ranging from 55° N to 45° S, was compiled from the global literature to test the hypothesis that post-dispersal seed removal by invertebrates and vertebrates is more intense at lower latitudes. We also quantified the relationship between post-dispersal seed removal by vertebrates and by invertebrates to global climatic features including temperature, actual evapotranspiration (AET) and rainfall seasonality. Linear mixed effect models were applied to describe the relationships between seed removal and latitude, hemisphere and climatic variables controlling for the effect of seed mass. Post-dispersal seed removal by invertebrates was negatively related to latitude. In contrast, post-dispersal seed removal by vertebrates was positively but weakly related to latitude. Mean annual temperature and actual evapotranspiration were positively related to post-dispersal seed removal by invertebrates, but not to post-dispersal seed removal by vertebrates, which was only marginally negatively related to rainfall seasonality. The inclusion of seed mass improved the fit of all models, but the term for seed mass was not significant in any model. Although a good climatic model for predicting post-dispersal seed predation by vertebrates at the global level was not found, our results suggest different and opposite latitudinal patterns of post-dispersal seed removal by invertebrates *vs* vertebrates. This is the first time that a negative relationship between post-dispersal seed removal by invertebrates and latitude, and a positive relationship with temperature and AET have been documented at a global-scale. These results have important implications for understanding global patterns in plant-animal interactions, and the factors that shape plant reproductive ecology, and also for predicting how this plant-animal interaction might respond to climate change.

## Introduction

The idea that biological interactions such as herbivory, predation and pathogen attack are more intense in the tropics is very widely accepted [1], [2]. This hypothesis is fundamental to theories explaining the latitudinal gradient in species richness [3]–[5] and has important implications for our understanding of latitudinal gradients in plant traits [1], [6]. However, tests for latitudinal gradients in herbivory [7], [8] and seed predation [9], [10] often do not support that interactions are more intense at lower latitudes.

Pre and post-dispersal seed predation can have huge impacts on plant populations when regeneration is seed limited [11], [12]. Seed predation also shapes seed traits [13], [14] and seed production strategies [15]. However, only two papers have quantified the relationship between seed predation and latitude. One of these papers [9] found no correlation between pre-dispersal seed predation and latitude for thirty-one populations of *Juniperus communis* in Europe. The other paper [10] found no significant relationship between post-dispersal seed removal and latitude (across 205 species from the global literature) or between pre-dispersal seed predation and latitude (across 138 species from the global literature). Nevertheless, both papers analyse seed removal without differentiating between animal guilds of different sizes.

Both invertebrates and vertebrates can be important seed predators [16], [17]. If these different guilds of seed predators respond to latitudinal gradients in environmental conditions differently, then they might display opposing latitudinal gradients in seed removal, potentially combining to generate no overall latitudinal gradient in seed removal. Invertebrates and vertebrates have different morphologies, physiologies and patterns of activity. Therefore, understanding global patterns in the importance of these two taxa will help us to understand global patterns in the factors shaping plant species' reproductive strategies, including seed mass, defences against seed predators, and temporal variability in seed production.

Variables such as temperature, actual evapotranspiration (AET) and rainfall seasonality are predicted to affect seed predation by vertebrates *vs* invertebrates in different, and often opposing ways.

There is a strong latitudinal gradient in land surface temperature, with mean annual temperature ranging from 30 °C at the equator to −50 °C in the Antarctic [18]. Since invertebrates are ectotherms, their activity might be limited by the low temperatures experienced at high latitudes, while the activity of vertebrate granivores, mostly endotherms, might be more independent of temperature [19], [20]. We therefore predict a positive relationship between temperature and seed removal by invertebrates, and no particular relationship between temperature and seed removal by vertebrates. Consistent with this prediction, positive relationships between air temperature and foraging activity [21], metabolic rate [22] and folivore density [23] have been reported for invertebrates.

Organisms may be limited by food availability and its predictability in time. According to the species-energy theory, energy availability regulates population sizes [24]–[26]. However, the number of individuals per unit energy will be lower if available energy varies through time than in a constant environment [27]. Actual evapotranspiration (AET), as an integrated measure of water-energy balance, has been used as a measure of total available energy and it is positively related with net primary productivity [25]. Intra-annual rainfall variability (henceforth rainfall seasonality), which is an indicator of seasonality in food availability [28], has been used as a measure of environmental unpredictability [29]. These variables are related to latitude in different ways, with AET being highest at low latitudes [27], but environmental predictability (the inverse of rainfall seasonality) being lowest at low latitudes [29]. There is evidence that endotherms have higher metabolic needs than do ectotherms by unit body mass [30] while ectotherms survive longer periods without feeding [31], [32]. Therefore, our working hypotheses are that AET will be positively related to seed removal by vertebrates, rainfall seasonality will be negatively related to seed removal by vertebrates, while seed removal by invertebrates will be more closely related to temperature (which drives energy assimilation [20]) and AET (which drives total available energy [25]) or rainfall seasonality (which drives unpredictability in food availability [28]. Our study will be the first to provide a global scale quantification of the effect of temperature, AET and rainfall seasonality on seed removal by vertebrates and invertebrates.

In summary our hypotheses were:

1. That there are opposite latitudinal gradients in seed removal rates by invertebrate and vertebrates...
2. That there will be a positive relationship between temperature and seed removal by invertebrates, and no relationship for vertebrates. Because of the negative relationship between temperature and latitude, we predict that the relationship between invertebrate seed removal and latitude will be negative.
3. That there will be a positive relationship between AET and seed removal by vertebrates and a negative relationship between rainfall seasonality and seed removal by vertebrates. Because these climatic variables are positively related to latitude, we predict that there will be no relationship between vertebrate seed removal and latitude.

## Methods

### Ethical statement

No permission or approval was required to obtain the data included in this study; all were taken from public sources.

### Data compilation

We searched for publications indexed in the ISI Web of Science from 1989 to February 2012 using the terms “removal” OR “predation” AND “seed” NOT “marine”. We found a total of 5343 papers, most of them with specific data on seed removal but without identification of the guilds responsible. From them we selected studies that provided paired-data of post-dispersal seed removal by vertebrates and invertebrates for different plant species in field exclusion experiments in 29 geographical sites. These experiments used seed removal trials using different selective exclosures for different animal guilds (ants-rodents-birds, vertebrates-invertebrates or others guild categories). Seed removal was used as an indication of seed predation because seed fate has rarely been measured. Nevertheless, we acknowledge that some of the seeds that are removed will not be consumed, and that seed removal can be an important vector of seed dispersal [33]. We excluded studies of frugivory and myrmecochory, and included only studies that measured removal of dry or clean seeds (no pulp or elaiosome), to exclude events of seed removal that are not likely to result in seed predation [33]. We only included studies of native species, because we aimed to analyze interactions where seed predators and the species they remove have had time to equilibrate to the study environment. We exclude studies on arable land or tree plantations because the intensive management and the abundance of commercial seeds could modify the natural patterns of seed predation. Post-dispersal seed removal data were averaged for all microenvironments, times and densities for each species in each locality. Under these criteria, we found paired data on seed removal by invertebrates and vertebrates for 79 plant species belonging to 39 families, occurring in a wide range of ecosystems and ranging from 55° N to 45° S (Appendix S1). The seed consumers included invertebrates (ants and beetles), and endothermic vertebrates (mammals and birds).

To obtain comparable seed removal data across studies of varying durations, we calculated the proportion of seeds removed by each guild after 24 h, assuming an exponential function (proportion of seeds removed on day 1 =  proportion of seed removed after n days^(1/n)^, see [34]). Exponential declines in seed removal through time have commonly been observed [35], [36]. When necessary, data were extracted from the graphs in the original papers using Datathief III [37]. The proportion of seed removal after 24 h ranged from 0 to 0.858 for invertebrates and from 0 to 0.480 for vertebrates. We also calculated the proportion of total seed removal due to invertebrates (removal by invertebrates over 24 h divided by removal by vertebrates over 24 hours).

Seed mass data were preferentially taken from the same paper as seed removal data, or from personal communications with the authors of these papers. For the remaining species, seed mass values were obtained from the Kew Seed Information Database [38]. Seed mass ranged from 0.33 to 30,000 mg (Appendix S1).

We recorded the latitude and longitude for each seed removal study. If geographical coordinates were not presented in the paper, they were taken from the nearest available location using Google Maps. Mean annual temperature and rainfall seasonality at 2.5 arc-minute resolution were obtained from the WorldClim 1.4 long term global climatic data base for the period 1950–2000 (available at: http://www.worldclim.org [39]). Mean annual evapotranspiration data (AET) were based on monthly averages for the period 1920–1980 at 0.5° resolution (available: http://www.grid.unep.ch/data/download/gnv183.zip, [40]).

### Statistical analysis

Seed removal data were logit transformed (ln(y/(1-y))) before analysis. Because the logit transformation is not possible for proportions of 0 or 1, we added or subtracted (for 0 and1 proportion values respectively) half of the minimum non-zero proportion to both the numerator and the denominator of the logit function [41].

We analyzed the data using linear-mixed effect models (GLMM, [42]), with seed removal (by invertebrates over 24 hours; by vertebrates over 24 hours; or the proportion of removal due to invertebrates) as the response variable. To quantify the effect of latitude we used absolute latitude as a fixed term in the models. To quantify the effect of climatic variables, we included mean annual temperature, mean annual AET or rainfall seasonality as fixed terms.

We included log seed mass as a covariate in both latitudinal and climatic models to investigate the possibility that significant relationships of predation rates with latitude or climatic variables were due to the positive relationship between seed mass and latitude [10], [6].

In all the models we included a random effect for site to account for site to site variation in the response variables not explained by the fixed terms. R^2^ for fixed and random effects in the models were calculated using sequential reduction in residual sum of squares in addition of the term but adding fixed effect terms in the model prior to the random-effects term. Models were fitted using restricted maximum likelihood with the Lme4Package [43] in R 3.0.1 [44].

## Results

### Post-dispersal seed removal *vs* latitude

**Figure 1:**
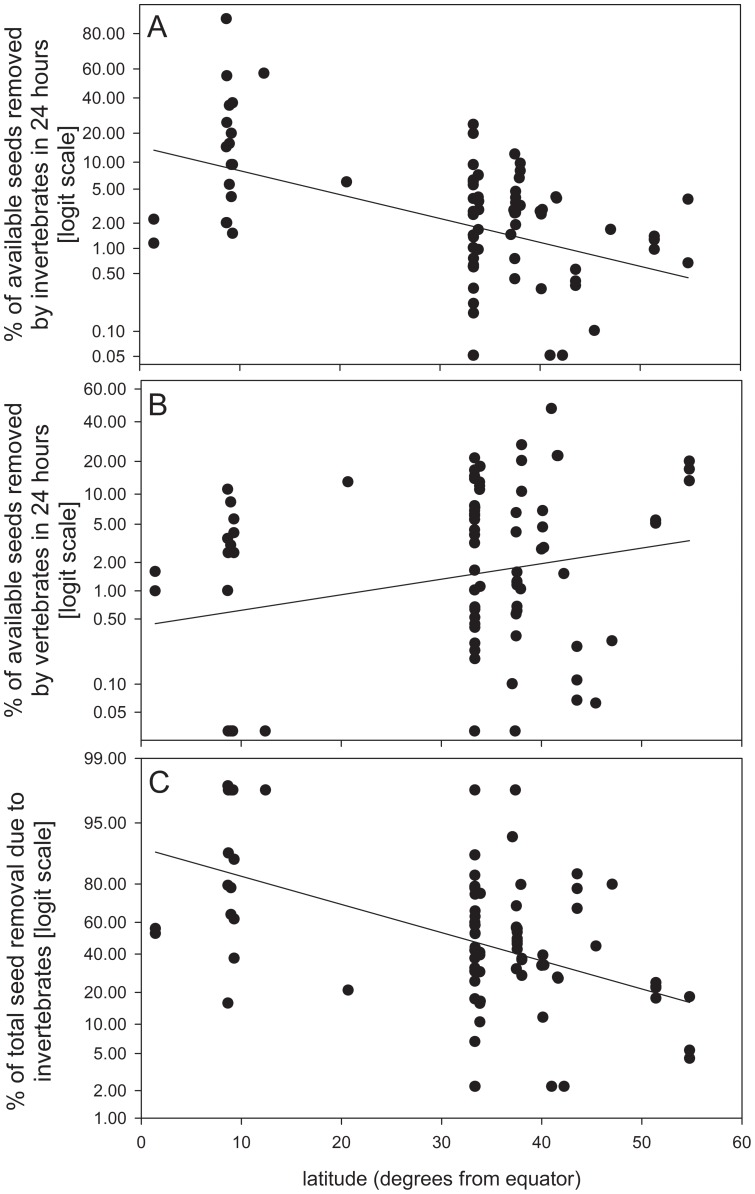
Percentage of available seeds removed in 24) invertebrates and b) vertebrates, and c) percentage of total seed removal due to invertebrates, in relation to latitude. The lines represent model predictions according with the model parameters specified in Table 1.

Post-dispersal seed removal by invertebrates was negatively related with latitude (*P* =  0.001, R^2^ = 0.18; Fig. 1a, Table 1). In contrast, post-dispersal seed removal by vertebrates was positively but weakly related to latitude (*P* = 0.03, R^2^ = 0.02; Fig. 1b, Table 1). The regression coefficient for seed mass was not significant in any of these models (P > 0.9 in all cases, Table 1).

**Table 1:** Summary of the mixed linear models fitted for the proportion of seed removed by invertebrates, vertebrates and proportion of total seed removal due to invertebrates in relation to absolute latitude.

Response variable	Explicative variables	Parameter	p-values	R^2^ fixed terms	R^2^ Within site
Proportion of seed removed by invertebrates.	Intercept	−1.770	0.003		
	Absolute latitude	−0.067	<0.001		
	Log seed mass	−0.010	0.963		
				0.185	0.613
Proportion of seed removed by vertebrates	Intercept	−5.579	<0.001		
	Absolute latitude	0.041	0.029		
	Log seed mass	0.016	0.948		
				0.076	0.479
Proportion of total seed removal due to invertebrates	Intercept	2.539	<0.001		
	Absolute latitude	−0.075	<0.001		
	Log seed mass	−0.150	0.470		
				0.246	0.578

The best models according to the AIC criteria included Log of seed mass in all the cases.

Invertebrates were responsible for an average of 47% (±32.5) of seed removal, and the proportion of total seed removal due to invertebrates was negatively related to latitude (*P*<0.0001, R^2^ = 0.25, Fig. 1c). The regression coefficient for seed mass was not significant (*P*>0.4 Table 1).

### Post-dispersal seed removal and climatic variables

**Figure 2:**
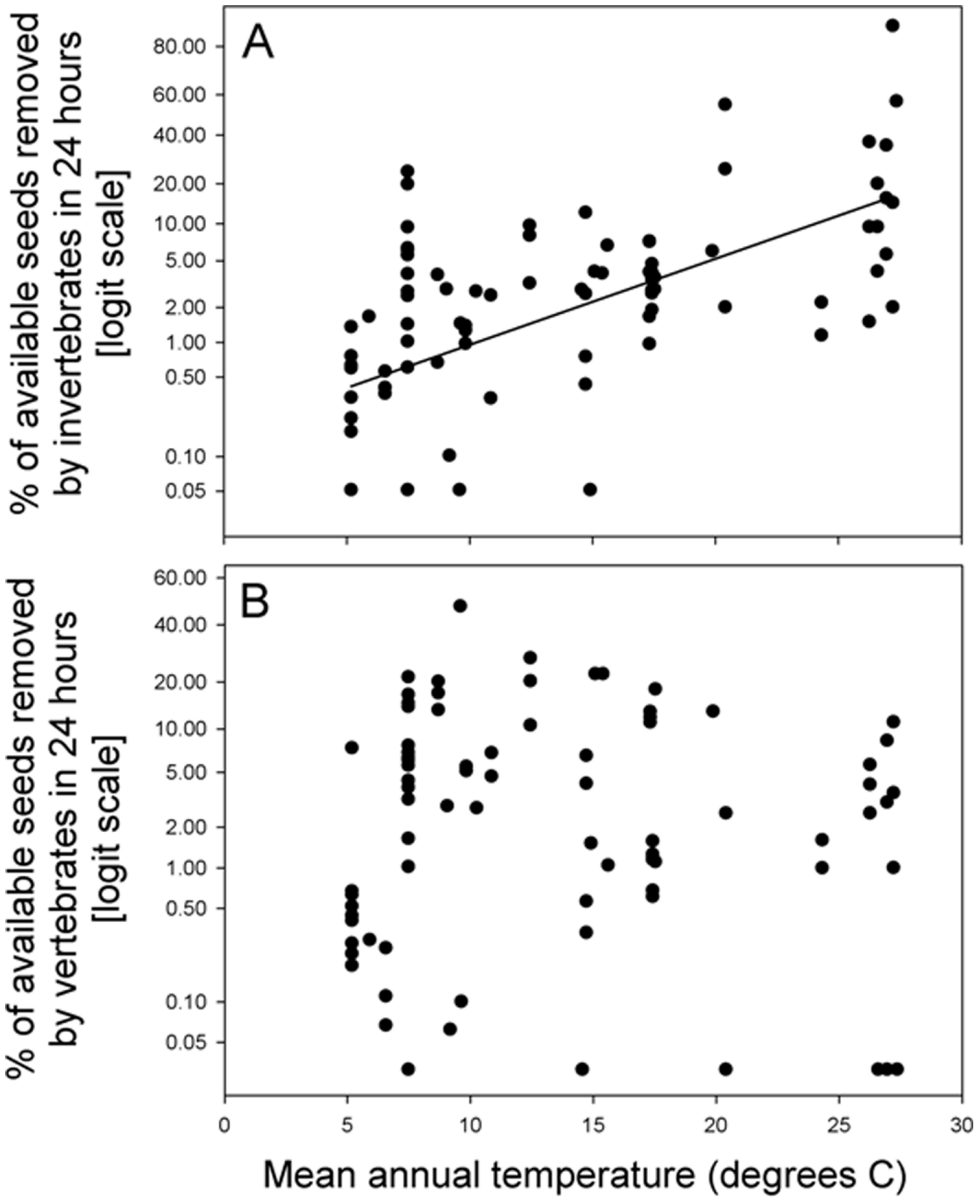
Percentage of available seeds removed in 24) invertebrates and b) vertebrates, in relation to mean annual temperature for the period 1950–2000 (climatic data from Hijmans et al. 2005, available at:http://www.worldclim.org). The line represents model predictions according with the model parameters specified in Table 2.

**Figure 3:**
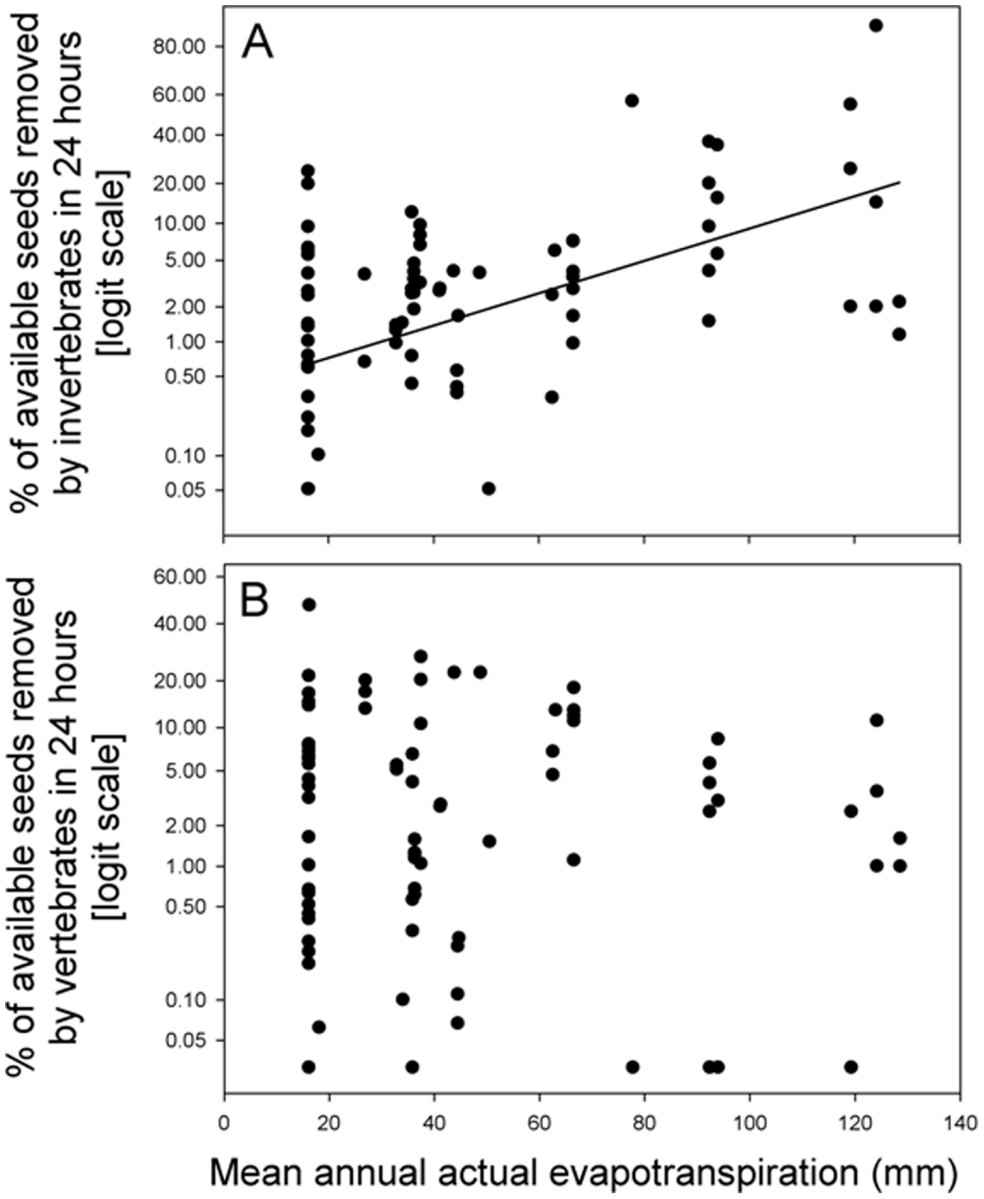
Percentage of available seeds removed in 24) invertebrates and b) vertebrates, in relation to mean annual AET for the for the period 1920–1980 (Ahn & Tateishi 1994, Tateishi & Ahn 1996, available at:http://www.grid.unep.ch/data/download/gnv183.zip). The line represents model predictions according with the model parameters specified in Table 2.

**Figure 4:**
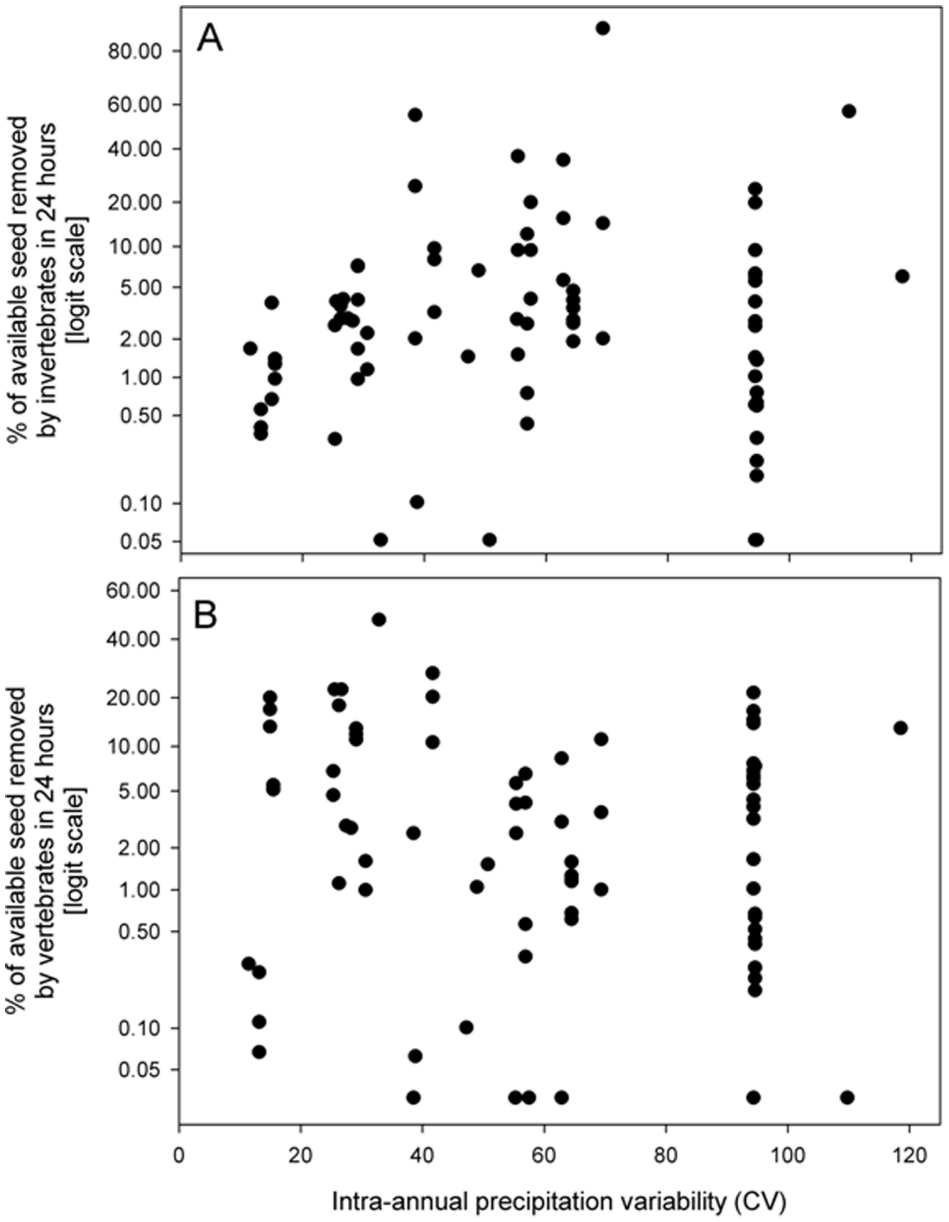
Percentage of available seeds removed in 24) invertebrates and b) vertebrates, in relation to rainfall seasonality (intra-annual precipitation variability (CV)) for the period 1950–2000 (climatic data from Hijmans et al. 2005, available at:http://www.worldclim.org).

Post-dispersal seed removal by invertebrates was positively related to mean annual temperature (*P*<0.0001, R^2^ = 0.42) (Fig. 2a, Table 2) and AET (*P*<0.0001, R^2^ = 0.30) (Fig. 3a) but not related to rainfall seasonality (*P* = 0.95) (Fig. 4a, Table 2). Post-dispersal seed removal by vertebrates was not related to mean annual temperature (*P* = 0.54, Fig. 2b, Table 2) or to AET (*P* =  0.45, Fig. 3b, Table 2), and only marginally negatively related to rainfall seasonality (*P* = 0.087, Fig. 4b, Table 2). The regression coefficient for seed mass was not significant in any of these models (P > 0.6 in all cases, Table 2).

**Table 2:** Summary of the mixed linear models fitted for the proportion of seed removed by invertebrates and vertebrates in relation to Mean annual Temperature, Mean annual evapotranspiration and Rainfall seasonality.

Response variable	Explicative variables	Parameter	p-values	R^2^ fixed terms	R^2^ Within site
Proportion of seed removed by invertebrates	Intercept	−6.335	<0.001		
	Mean annual temperature	0.173	<0.001		
	Log seed mass	−0.078	0.670		
				0.422	0.578
Proportion of seed removed by invertebrates	Intercept	−5.557	<0.001		
	AET	0.033	<0.001		
	Log seed mass	−0.045	0.816		
				0.304	0.696
Proportion of seed removed by invertebrates	Elevation	−3.747	<0.001		
	Rainfall seasonality	−0.001	0.947		
	Log seed mass	−0.039	0.866		
				0.036	0.590
Proportion of seed removed by vertebrates	Intercept	−3.947	<0.001		
	Mean annual temperature	−0.025	0.535		
	Log seed mass	0.017	0.946		
				0.007	0.465
Proportion of seed removed by vertebrates	Intercept	−3.972	<0.001		
	AET	−0.006	0.451		
	Log seed mass	0.012	0.960		
				0.010	0.466
Proportion of seed removed by vertebrates	Intercept	−3.340	<0.001		
	Rainfall seasonality	−0.018	0.087		
	Log seed mass	−0.020	0.935		
				0.030	0.463

AET: Mean annual evapotranspiration.

The best models according to the AIC criteria included Log of seed mass in all the cases.

## Discussion

### Is there a latitudinal gradient in post-dispersal seed removal?

Our results show different and opposite patterns of post-dispersal seed removal by invertebrates *vs* vertebrates in relation to latitude. While seed removal by invertebrates decreases as latitude increases, seed removal by vertebrates increases. Our results only partially support the hypothesis that biological interactions should be higher in the tropics [3], [45] and can explain the lack of relationship between post-dispersal seed predation and latitude reported by Moles *et al.* [10].

We found that invertebrates were responsible for 47% of the total seed removal. That is, the data so far suggest that vertebrates and invertebrates are approximately equally important for seeds. Surprisingly, this is only the second time anyone has quantified overall importance of vertebrates *vs* invertebrates for seed removal. Hulme [13] found that rodents were responsible for almost twice as much seed predation (45%) as were invertebrates (24%), but acknowledged that a lack of data from the southern hemisphere may have resulted in an underestimate of the importance of invertebrates. Invertebrates and vertebrates have very different morphologies and physiologies, including differences in body size, behaviour, mouthparts, digestion and thermoregulation. Thus, knowing the relative impact of vertebrates and invertebrates on seeds will help us to better understand the selective forces that have shaped physical and chemical defences of seeds, and how different climatic variables and habitat characteristics shape larger biogeographic patterns in seed predation. Gathering data on the relative importance of vertebrates and invertebrates for other types of plant-animal interaction (such as herbivory) is an important goal for the future.

We found that the percentage of total seed removal due to invertebrates decreases with latitude, due to both a decrease in seed removal by vertebrates with increasing latitude, and an increase in seed removal by invertebrates. This pattern could have important consequences for understanding the evolution of seed traits because plants may be specialized in defending their seeds against different guilds of granivores in different regions of the globe. For example, smooth spherical seeds have been reported to be avoided by ants while birds avoid awned seeds [46]. Another possibility is that the seed size specialization between ants and mammals [19] might contribute to the latitudinal gradient in seed size [6].

### Can we predict rates of seed removal with simple climatic variables?

One very important finding of this study, which has not been reported until now, is that invertebrate seed removal can be relatively well predicted (42% of the total variation) by mean annual temperature at the global level, although there is still room for explanatory variables that act at the within-site level. In contrast, seed removal by vertebrates was independent of mean annual temperature. These results are consistent with the Energetic-equivalence rule proposed by Allen *et al*. [20]. This rule predicts an inverse relationship between population density and temperature for ectotherms (both plants and animals, because of their similar activation energy for metabolism) and no relationship for endotherms after body mass correction [20], [47], [48].

We predicted that AET (an indicator of food availability) and rainfall seasonality (an indicator of environmental variability) would be positively related to seed removal by vertebrates, while seed removal by invertebrates would be more strongly related to temperature (which has been shown to limit rates of energy assimilation [20]). These predictions have been only partially supported. Invertebrate seed removal was positively related to AET, but was even more strongly related to temperature. Seed removal by vertebrates was negatively (but weakly) related to rainfall seasonality. This result might be related to the reproductive and demographic constraints imposed on bird and mammal populations by seasonality in resource availability [28], [48]. However, contrary to our expectations, seed removal by vertebrates was not related to AET although there is a positive relationship between AET and species richness and population density in mammals and birds [27].

The relatively low explanatory power of climatic variables in relation to vertebrate seed removal could be due to the high mobility of vertebrates, especially in the case of migrant species that can avoid seasons of low food availability [49]. Alternatively, vertebrate seed removal activity might be more dependent on local factors such as the presence of shrubs or trees which act as refuges minimizing the predation risk [50]–[53] than on broad scale climatic variables.

### Data limitations

Unfortunately, we found very few experimental studies of seed removal in the south hemisphere that fit our requirements, all concentrated in a narrow latitudinal band. This prevented an analysis of the effect of hemisphere on the observed trends. We also included few studies from deserts in the present paper because most studies of seed removal by different guilds in desert ecosystems have used highly palatable commercial seeds such as canary grass and millet, which we excluded as they often have artificially high rates of seed removal [12]. A major direction to take in future research will be the improvement of our understanding of the relative importance of seed removal by vertebrates and invertebrates of native plant species in natural ecosystems, along a wide range of latitudes in both hemispheres, as this information will shed light on an important selective process that shape plant reproduction in seed limited plant species.

### A general conclusion

In summary, we documented a negative relationship between post-dispersal seed removal by invertebrates and latitude at the global-scale. We also provided the first quantification of the associations between seed removal and three crucial climatic variables, temperature, rainfall seasonality and AET. Our results have important consequences for understanding global patterns in seed removal, and the factors that shape plant reproductive ecology, and also for predicting how this plant-animal interaction might respond to climate change.

## Supporting Information

Appendix S1 Database of proportion of post-dispersal seed removal by vertebrates (ver_24h) and invertebrates (inver_24h) after 24 hours of exposure, used for the global analysis. Species names, seed mass (mg), type of granivores, absolute latitude, study location and reference are also included. NA: Not available.
(DOCX)
