# Supplementary material for: Global Patterns in Post-Dispersal Seed Removal by Invertebrates and Vertebrates
Source: PLoS One. 2014 Mar 11;9(3):e91256. doi: 10.1371/journal.pone.0091256 (PMC3949765; doi:10.1371/journal.pone.0091256)
Supplement: Appendix S1 — Database of proportion of post-dispersal seed removal by vertebrates (ver_24h) and invertebrates (inver_24h) after 24 hours of exposure, used for the global analysis. Species names, seed mass (mg), type of granivores, absolute latitude, study location and reference are also included. NA: Not available. (DOCX) [file pone.0091256.s001.docx]

**SUPPORTING INFORMATION**

**Appendix S1** Database of proportion of post-dispersal seed removal by vertebrates (ver_24h) and invertebrates (inver_24h) after 24 hours of exposure, used for the global analysis. Species names, seed mass (mg), type of granivores, absolute latitude, study location and reference are also included. NA: Not available.

| Reference | Location | Latitude | Species | Seed mass | Granivores | inv_24h | ver_24h |
| --- | --- | --- | --- | --- | --- | --- | --- |
| [1] | Argentina | -45.42 | *Mulinum spinosum* | 5.4 | ants, rodents, birds | 0.001 | 0.001 |
| [2] | Spain | 41.58 | *Pinus halepensis* | 23.0 | ants, rodents | 0.041 | 0.223 |
| [3] | USA | 41.00 | 3 native spp | NA | ants,rodents, birds | 0.000 | 0.480 |
| [4] | Spain | 37.56 | *Pinus contorta* | 4.0 | ants, rodents, birds | 0.035 | 0.016 |
| [4] | Spain | 37.56 | *Pinus patula* | 9.0 | ants, rodents, birds | 0.047 | 0.012 |
| [4] | Spain | 37.56 | *Pinus halepensis* | 17.0 | ants, rodents, birds | 0.040 | 0.013 |
| [4] | Spain | 37.56 | *Pinus coulteri* | 347.0 | ants rodentsbirds | 0.028 | 0.012 |
| [4] | Spain | 37.56 | *Pinus pinea* | 620.0 | ants rodentsbirds | 0.019 | 0.006 |
| [4] | Spain | 37.56 | *Pinus sabiniana* | 829.0 | ants rodentsbirds | 0.027 | 0.007 |
| [5] | Mexico | 20.67 | *Opuntia rastrera* | NA | birds, ants rodent | 0.060 | 0.131 |
| [6] | Spain | 42.25 | *Corema album* | 30.1 | invertebrates, vertebrates | 0.000 | 0.015 |
| [7] | Spain | 37.38 | *Erysimum mediohispanicum* | 0.4 | ants, beetles, all predators | 0.029 | 0.000 |
| [7] | Spain | 37.08 | *Erysimum baeticum* | NA | ants, beetles, all predators | 0.015 | 0.001 |
| [8] | UK | 54.77 | *Fraxinus excelsior* | 29.3 | invertebrates, vertebrates | 0.007 | 0.134 |
| [8] | UK | 54.77 | *Taxus baccata* | 69.9 | invertebrates, vertebrates | 0.007 | 0.171 |
| [8]) | UK | 54.77 | *Ulmus glabra* | 3.5 | invertebrates, vertebrates | 0.038 | 0.201 |
| [9] | Spain | 37.47 | *Crataegus monogyna* | 193.0 | ants, rodents | 0.008 | 0.006 |
| [9] | Spain | 37.47 | *Hedera helix* | 23.6 | ants, rodents | 0.123 | 0.042 |
| [9] | Spain | 37.47 | *Smilax aspera* | 43.7 | ants, rodents | 0.004 | 0.003 |
| [9] | Spain | 37.47 | *Olea europaea* | 104.8 | ants, rodents | 0.026 | 0.066 |
| [9] | Spain | 38.02 | *Crataegus monogyna* | 140.0 | ants, rodents | 0.032 | 0.106 |
| [9] | Spain | 38.02 | *Berberis hispanica* | 23.7 | ants, rodents | 0.081 | 0.203 |
| [9] | Spain | 38.02 | *Rosa spp* | NA | ants, rodents | 0.097 | 0.274 |
| [10] | UK | 51.41 | *Arrhenatherum elatius* | 2.2 | invertebrates, rodents | 0.014 | 0.051 |
| [10] | UK | 51.41 | *Centaurea nigra* | 2.5 | invertebrates, rodents | 0.013 | 0.055 |
| [10] | UK | 51.41 | *Festuca rubra* | 0.7 | invertebrates, rodents | 0.013 | 0.052 |
| [10]) | UK | 51.41 | *Rumex acetosa* | 0.8 | invertebrates, rodents | 0.010 | 0.055 |
| [11] | Australia | -33.88 | *Grevillea longifolia* | 127.0 | mamals, invertebrates | 0.036 | 0.011 |
| [11] | Australia | -33.88 | *Grevillea caleyi* | 331.0 | mamals, invertebrates | 0.029 | 0.181 |
| [12] | Chile | -33.35 | *Chuquiraga oppositifolia* | 7.5 | ants, birds | 0.014 | 0.004 |
| [12] | Chile | -33.35 | *Arthrophyllum cumingii* | 23.6 | ants, birds | 0.000 | 0.074 |
| [12] | Chile | -33.35 | *Taraxacum officinale* | 0.3 | ants, birds | 0.006 | 0.004 |
| [12] | Chile | -33.35 | *Rhodophiala rhodolirion* | 17.2 | ants, birds | 0.006 | 0.005 |
| [12] | Chile | -33.35 | *Alstroemeria pallida* | 25.6 | ants, birds | 0.002 | 0.002 |
| [12] | Chile | -33.35 | *Sisyrinchium arenarium* | 6.5 | ants, birds | 0.002 | 0.003 |
| [12] | Chile | -33.35 | *Laretia acaulis* | 9.2 | ants, birds | 0.014 | 0.006 |
| [12] | Chile | -33.35 | *Azorella monantha* | 0.8 | ants, birds | 0.008 | 0.007 |
| [12] | Chile | -33.35 | *Pozoa coriacea* | 2.6 | ants, birds | 0.003 | 0.002 |
| [13] | USA | 40.11 | *Cornus drummondii* | 50.4 | birds, ants, mammals | 0.026 | 0.069 |
| [13] | USA | 40.11 | *Prunus serotina* | 83.9 | Invertebrates, vertebrates | 0.003 | 0.047 |
| [14] | Switzerland | 47.05 | *Picea abies* | 7.0 | invertebrates, rodents, cows, birds | 0.017 | 0.003 |
| [15] | Spain | 41.66 | *Pinus halepensis* | 23.0 | ants, rodents | 0.039 | 0.223 |
| [16] | Chile | -33.33 | *Acaena alpina* | 70.2 | insect, birds, rodents | 0.006 | 0.010 |
| [16] | Chile | -33.33 | *Acaena pinnatifida* | 8.9 | insect, birds, rodents | 0.006 | 0.000 |
| [16] | Chile | -33.33 | *Adesmia microphylla* | 5.9 | insect, birds, rodents | 0.025 | 0.064 |
| [16] | Chile | -33.33 | *Alstroemeria ligtu* | 19.1 | insect,bird rodents | 0.000 | 0.000 |
| [16] | Chile | -33.33 | *Alstroemeria pallida* | 16.5 | insect, birds, rodents | 0.000 | 0.000 |
| [16] | Chile | -33.33 | *Aristotelia chilensis* | 76.6 | insect, birds, rodents | 0.242 | 0.044 |
| [16] | Chile | -33.33 | *Colliguaja integerrima* | 213.5 | insect, birds, rodents | 0.000 | 0.010 |
| [16] | Chile | -33.33 | *Eccremocarpus scaber* | 0.4 | insect, birds, rodents | 0.000 | 0.061 |
| [16] | Chile | -33.33 | *Galium eriocarpum* | 3.2 | insect, birds, rodents | 0.000 | 0.017 |
| [16] | Chile | -33.33 | *Guindilla trinervis* | 533.8 | insect, birds, rodents | 0.039 | 0.010 |
| [16] | Chile | -33.33 | *Kageneckia oblonga* | NA | insect, birds, rodents | 0.000 | 0.056 |
| [16] | Chile | -33.33 | *Leucocoryne ixioides* | 3.1 | insect, birds, rodents | 0.027 | 0.069 |
| [16] | Chile | -33.33 | *Loasa postrata* | 12.4 | insect, birds, rodents | 0.199 | 0.017 |
| [16] | Chile | -33.33 | *Loasa sp* | 13.1 | insect, birds, rodents | 0.000 | 0.039 |
| [16] | Chile | -33.33 | *Madia sativa* | 3.8 | insect, birds, rodents | 0.000 | 0.069 |
| [16] | Chile | -33.33 | *Malesherbia linearifolia* | 1.6 | insect, birds, rodents | 0.014 | 0.077 |
| [16] | Chile | -33.33 | *Mutisia ilicifolia* | 113.0 | insect, birds, rodents | 0.000 | 0.000 |
| [16] | Chile | -33.33 | *Oxalis sp* | 0.9 | insect, birds, rodents | 0.010 | 0.214 |
| [16] | Chile | -33.33 | *Ribes polyanthes* | NA | insect, birds, rodents | 0.056 | 0.010 |
| [16] | Chile | -33.33 | *Schinus montanus* | 47.2 | insect, birds, rodents | 0.056 | 0.039 |
| [16] | Chile | -33.33 | *Sisyrinchium arenarium* | 4.6 | insect,bird rodents | 0.010 | 0.032 |
| [16] | Chile | -33.33 | *Sisyrinchium philippii* | 5.0 | insect, birds, rodents | 0.000 | 0.148 |
| [16] | Chile | -33.33 | *Stachys albicaulis* | 2.1 | insect, birds, rodents | 0.064 | 0.167 |
| [16] | Chile | -33.33 | *Trevoa quinquenervia* | 10.4 | insect, birds, rodents | 0.061 | 0.039 |
| [16] | Chile | -33.33 | *Tetraglochin alatum* | 6.1 | insect, birds, rodents | 0.000 | 0.056 |
| [16] | Chile | -33.33 | *Tristerix verticillatus* | 157.7 | insect, birds, rodents | 0.094 | 0.140 |
| [16] | Chile | -33.33 | *Tropaeolum tricolor* | 23.5 | insect, birds, rodents | 0.000 | 0.000 |
| [17] | Spain | 40.01 | *Pinus nigra subsp salzmannii* | 16.4 | ants, birds, rodents | 0.028 | 0.028 |
| [17] | Spain | 40.23 | *Pinus nigra subsp salzmannii* | 16.4 | ants, birds, rodents | 0.029 | 0.029 |
| [18] | Panama | 8.97 | *Apeiba aspera* | 14.2 | invertebrates,vertebrates | 0.157 | 0.000 |
| [18] | Panama | 8.97 | *Cecropia peltata* | 0.5 | ants, vertebrates | 0.352 | 0.083 |
| [18] | Panama | 8.97 | *Luehea seemannii* | 1.0 | ants, vertebrates | 0.057 | 0.030 |
| [18] | Panama | 9.17 | *Apeiba aspera* | 14.2 | ants, vertebrates | 0.200 | 0.000 |
| [18] | Panama | 9.17 | *Cecropia peltata* | 0.5 | ants, vertebrates | 0.094 | 0.000 |
| [18] | Panama | 9.17 | *Luehea seemannii* | 1.0 | ants, vertebrates | 0.041 | 0.000 |
| [18] | Panama | 9.28 | *Apeiba aspera* | 14.2 | ants, vertebrates | 0.368 | 0.041 |
| [18] | Panama | 9.28 | *Cecropia peltata* | 0.5 | ants, vertebrates | 0.094 | 0.057 |
| [18] | Panama | 9.28 | *Luehea seemannii* | 1.0 | ants, vertebrates | 0.015 | 0.025 |
| [18] | Panama | 8.67 | *Apeiba aspera* | 14.2 | ants, vertebrates | 0.859 | 0.010 |
| [18] | Panama | 8.67 | *Cecropia peltata* | 0.5 | ants, vertebrates | 0.146 | 0.036 |
| [18] | Panama | 8.67 | *Luehea seemannii* | 1.0 | ants, vertebrates | 0.020 | 0.111 |
| [18] | Panama | 8.72 | *Apeiba aspera* | 14.2 | ants, vertebrates | 0.553 | 0.000 |
| [18] | Panama | 8.72 | *Cecropia peltata* | 0.5 | ants, vertebrates | 0.252 | 0.025 |
| [18] | Panama | 8.72 | *Luehea seemannii* | 1.0 | ants, vertebrates | 0.020 | 0.000 |
| [19] | USA | 43.55 | *Medicago lupulina* | 1.6 | ants, rodents, birds | 0.004 | 0.001 |
| [19] | USA | 43.55 | *Centaurea nigra* | 2.2 | ants, rodents, birds | 0.006 | 0.003 |
| [19] | USA | 43.55 | *Taraxacum officinale* | 0.7 | ants, rodents, birds | 0.004 | 0.001 |
| [20] | Australia | -33.83 | *Allocasuarina distyla* | 3.4 | ants, small mamals | 0.073 | 0.120 |
| [20] | Australia | -33.83 | *Banksia serrata* | 76.9 | ants, small mamals | 0.017 | 0.120 |
| [20] | Australia | -33.83 | *Banksia spinulosa* | 11.4 | ants, small mamals | 0.072 | 0.130 |
| [20] | Australia | -33.83 | *Hakea gibbosa* | 67.8 | ants, small mamals | 0.010 | 0.111 |
| [20] | Australia | -33.83 | *Hakea teretifolia* | 13.6 | ants, small mamals | 0.040 | 0.120 |
| [21] | Australia | -12.42 | *Sorghum intrans* | 13.0 | ants, vertebrates | 0.570 | 0.000 |
| [22] | Spain | 37.93 | *Pistacia terebinthus* | 38.0 | ants, vertebrates | 0.067 | 0.010 |
| [23] | Zaire | 1.42 | *Gilbertiodendron dewevrei* | 30000.0 | invertebrates, antelopes, rodents | 0.022 | 0.016 |
| [23] | Zaire | 1.42 | *Julbernardia seretii* | 4000.0 | invertebrates, antelopes, rodents | 0.012 | 0.010 |

**REFERENCES**

1 Folgareit PJ, Sala OE (2002) Granivory rates by rodents, insects and birds at different microsites in the Patagonian steppe. Ecography 25: 417-427.

2 Broncano MJ, Retama J, Rodrigo A (2006) Predicting the recovery of Pinus halepensis and Quercus ilex forest after a large wildfire in northeastern Spain. Plant Ecol 180: 47-56

3 Parmenter RR, MacMahon JA, Vander Wall SB (1984) The measurement of granivory by desert rodents, birds and ants: a comparison of an energetics approach and a seed-dish technique. J Arid Environ 7: 75-92.

4 Carrillo-Gavilán MA, Lalagüe H, Villa M (2010) Comparing seed removal of 16 pine species differing in invasiveness. Biol inv 12: 2233-2242.

5 Montiel S, Montaña C (2003) Seed bank dynamics of the desert cactus Opuntia rastrera in two habitats. Plant Ecol 166: 241-248.

6 Calviño-Cancela M (2007) Seed and microsite limitations of recruitment and the impacts of post-dispersal seed predation at the within population level. Plant Ecol 192: 35-44.

7 Gomez JM (2005) Long-Term Effects of Ungulates on Performance, Abundance, and Spatial Distribution of Two Montane Herbs. Ecol Mono 75: 231-258.

8 Hulme PH, Borrelli T (1999) Variability in Post-Dispersal Seed Predation in Deciduous Woodland: Relative Importance of Location, Seed Species, Burial and Density. Plant Ecol 145: 149-156.

9 Rey PJ, Garrido JL, Alcántara JM, Ramirez JM, Aguilera A, García L, Manzaneda AJ, Fernández R (2002) Spatial variation in ant and rodent post-dispersal seed predation of vertebrates dispersed seeds. Funct Ecol 16: 773-781.

10 Edwards GR, Crawley MJ (1998) Rodent seed predation and seedling recruitment in mesic grassland. Oecologia 118: 288 -296.

11 Auld TD, Denham AJ (1999) The Role of Ants and Mammals in Dispersal and Post-Dispersal Seed Predation of the Shrubs Grevillea (Proteaceae). Plant Ecol 144: 201-213.

12 Muñoz AA, Cavieres LA (2006) A Multi-species Assessment of Post-dispersal Seed Predation in the Central Chilean Andes. Ann Bot 98: 193-201.

13 Whelan CJ, Willson MF, Tuma CA, Souza-Pinto I (1991) Spatial and temporal patterns of postdispersal seed predation. Canadian J Bot 69: 428-436.

14 Smith C, Gusberti M, Müller-Schärer H (2006) Safe for saplings: safe for seeds? Forest Ecol Mang 237: 471-477.

15 Broncano MJ, Rodrigo A, Retama J (2008) Postdispersal seed predation in Pinus halepensis and consequences on seedling establishment arter fire. Int J Wildland fire 17: 407-414.

16 Figueroa, J.A., Muñoz,A. J. & Arroyo, M.T.K. (2002). Pre- and past-dispersal seed predation in a mediterranean-type climate montane sclerophyllus forest in Central Chile. Aus J Bot 50: 183-195.

17 Lucas-Borja ME, Silva-Santos P, Fonseca TF, Tíscar Oliver P A, López Serrano FR, Andrés Abellán M, Martínez García E, del Cerro Barja A (2010) Modelling Spanish black pine postdispersal seed predation in Central-eastern Spain. Forest Syst 19: 393-403.

18 Fornara DA, Dalling JW (2005) Post-Dispersal Removal of Seeds of Pioneer Species from Five Panamanian Forests. J Trop Ecol 21: 79-84.

19 Reader RJ, Beisner BE (1991) Species-dependent Effects of Seed Predation and Ground Cover on Seedling Emergence of Old-field Forbs. Am Midl Nat 126: 279-286.

20 Tasker EM, Denham AJ, Taylor JE, Strevens TC (2011) Post-fire seed predation: Does distance to unburnt vegetation matter?. Austra Ecol 36: 755–766

21 Andrew MH (1986) Granivory of the Annual Grass Sorghum intrans by the Harvester Ant Meranoplus sp. inTropical. Biotropica 18: 344-349.

22 Traveset A (1994) Cumulative Effects on the Reproductive Output of Pistacia terebinthus (Anacardiaceae). Oikos 71: 152-162.

23 Hart TB (1995) Seed, seedling and sub-canopy survival in monodominant and mixed forest of the Ituri Forest, Africa. J Trop Ecol 11: 443-459.
